# Step-by-step variability of swing phase trajectory area during steady state walking at a range of speeds

**DOI:** 10.1371/journal.pone.0191247

**Published:** 2018-01-25

**Authors:** Deanna D. Rumble, Christopher P. Hurt, David A. Brown

**Affiliations:** 1 PhD in Rehabilitation Science Program, University of Alabama at Birmingham, Birmingham, Alabama, United States of America; 2 Department of Physical Therapy, University of Alabama at Birmingham, Birmingham, Alabama, United States of America; Northwestern University, UNITED STATES

## Abstract

**Background:**

Step kinematic variability has been characterized during gait using spatial and temporal kinematic characteristics. However, people can adopt different trajectory paths both between individuals and even within individuals at different speeds. Single point measures such as minimum toe clearance (MTC) and step length (SL) do not necessarily account for the multiple paths that the foot may take during the swing phase to reach the same foot fall endpoint. The purpose of this study was to test a step-by-step foot trajectory area (SBS-FTA) variability measure that is able to characterize sagittal plane foot trajectories of varying areas, and compare this measure against MTC and SL variability at different speeds. We hypothesize that the SBS-FTA variability would demonstrate increased variability with speed. Second, we hypothesize that SBS-FTA would have a stronger curvilinear fit compared with the CV and SD of SL and MTC. Third, we hypothesize SBS-FTA would be more responsive to change in the foot trajectory at a given speed compared to SL and MTC. Fourth, SBS-FTA variability would not strongly co-vary with SL and MTC variability measures since it represents a different construct related to foot trajectory area variability.

**Methods:**

We studied 15 nonimpaired individuals during walking at progressively faster speeds. We calculated SL, MTC, and SBS-FTA area.

**Results:**

SBS-FTA variability increased with speed, had a stronger curvilinear fit compared with the CV and SD of SL and MTC, was more responsive at a given speed, and did not strongly co-vary with SL and MTC variability measures.

**Conclusion:**

SBS foot trajectory area variability was sensitive to change with faster speeds, captured a relationship that the majority of the other measures did not demonstrate, and did not co-vary strongly with other measures that are also components of the trajectory.

## Background

Step kinematic variability has been characterized during gait using spatial and temporal kinematic characteristics such as step length, step height, step time, and cadence [1–4]. Particularly when studied at faster speeds, step kinematic variability can provide insight into limb trajectory control that might lead to gait transitions or even loss of control. The literature on the nature of the relationship between speed and step kinematic variability describes different observations, which may be partially due to how variability is measured. For example, Sekiya et. al. [3] observed that, for speeds less or greater than their comfortable walking speed (CWS), step length (SL) variability (i.e. standard deviation) of nonimpaired individuals was increased. In contrast, Jordan et. al. [5] and Chien et. al. [6] observed that there is a small decline in coefficient of variation (CV) with SL at 80% CWS compared to fast walking speeds which would indicate a reduction in the magnitude of the variability. Osaki [7] demonstrated that the relative position coordinates of the foot at toe-off and heel contact is strongly influenced by walking speed, but that toe clearance is minimally affected by walking speed. Others have shown that variability of minimum toe clearance (MTC) typically increases with speed [8, 9] and, when measured at self-selected gait speeds individuals with slower CWS, have greater variability than individuals with faster CWS [2, 10–12].

Past studies have demonstrated that foot trajectory variability, between individuals that walk more slowly compared to individuals that do not walk as slowly, can be an important marker for reduced CWS and increased fall risk. For example, greater variability of step kinematics is associated with decreased gait stability and increased risk of falls in fall risk populations [2, 3, 10, 11, 13–16]. Therefore, with this study, we sought to investigate measures of foot path variability at a range of speeds so as to reveal the sensitivity of each measure to changes associated with footpath control. The foot trajectory of individuals while walking is not fixed to a prescribed path between individuals or across a range of speeds within the same individual. Thus, people can adopt different trajectory paths both between individuals and even within individuals at different speeds. For non-impaired populations walking at a comfortable speed, the trajectory of the foot maintains a continuous, repeatable pattern with some cycle-to-cycle (i.e., step-to-step) variations. While minimum toe clearance and step length provide information about points along that trajectory, they do not provide information on the variations of the continuous trajectory of the foot on a step-to-step basis. The full trajectory of the foot provides insight on the end point control of this multi-linked segment. Control of this end point by the nervous system can be affected by contextual factors like neurological impairment (i.e., stroke) or scaling factors like walking speed. In fact, cycle-to-cycle variations in movement of a segment may provide more sensitive information that relates to the overall stability of the movement pattern [17]. The full trajectory of biomechanical movement has been used by others using nonlinear tools that focus that how a movement pattern changes over time by assuming that each cycle of movement is not independent of past and future cycles [18]. However, this analysis requires a large sample of steps [19, 20], which is difficult in individuals with gait impairments particularly at faster speeds, to calculate a single variable that describes the overall variability and structure of variability of the behavior, and does not analyze on a step-by-step basis.

We sought to capture the bi-dimensionality of foot trajectory in the sagittal plane by a curvilinear approach into an area measurement on a step-by-step basis. Therefore, the purpose of this study was to test a step-by-step foot trajectory area (SBS-FTA) variability measure that is able to characterize foot trajectories of varying areas, and compare this measure against MTC and SL variability at different speeds. The SBS-FTA variable was quantified by measuring step-by-step changes in the area under the toe marker during swing phase (H_T_). Further, to capture differences in initial swing phase versus terminal swing, we divided that area measurement into two regions from foot off to midswing (H_1_), and midswing to initial foot contact (H_2_). The foot trajectory sub-phases between individuals are relatively consistent for healthy adults. However, we divided by these sub-phases because part of this current study is to establish a metric using healthy controls for individuals where these sub-phases are less consistent (chronic poststroke hemiparesis).

With this paper we assessed which measure of variability was most responsive to change across a range of speeds. The types of variability responsiveness tested were SL variability and MTC variability using standard deviation (SD) and CV, and SBS-FTA variability. We hypothesize that the SBS-FTA variability would demonstrate increased variability with speed. Second, we hypothesize that SBS-FTA would have a stronger curvilinear fit compared with the CV and SD of SL and MTC. Third, we hypothesize SBS-FTA would be more responsive to change in the foot trajectory at a given speed compared to SL and MTC. Fourth, that SBS-FTA variability would not strongly co-vary with SL and MTC variability measures since it represents a different construct related to foot trajectory area variability. This can provide more information about how this endpoint control is modified by some scaling factor on a step-by-step basis, like walking speed, and may provide information that can be beneficial to study impaired populations whose endpoint control is affected.

## Methods

### Participants

We recruited fifteen non-impaired individuals (age: 57.0±16.0, 8 female). Inclusion criteria were: older than 19 years of age, ability to walk independently, medically stable, and able to provide written informed consent. Exclusion criteria were: history of serious medical conditions, uncontrolled respiratory or metabolic disorders, uncontrolled blood pressure (systolic pressure >180 mmHg, diastolic blood pressure >110 mmHg), presence of cerebellar and brainstem deficits, severe cognitive disorder, inability to follow simple commands, and major or acute musculoskeletal problems. This study was performed at the University of Alabama at Birmingham (UAB) and written informed consent was obtained from all participants. This study was approved by UAB’s Institutional Review Board under protocol number: F141212010. The study was conducted in accordance with the principles of the latest version of the Declaration of Helsinki. Recruitment and experimental procedures were completed by the investigators.

### Materials

Passive reflective markers were placed bi-laterally on the feet on the second and fifth metatarsal heads, lateral ankle malleoli, and the calcaneus and were collected by an 8 camera Qualisys motion capture system sampling at 100Hz. Marker positions were tracked using QTM 2.2 offline. Marker position data was processed via Visual3D software (C-motion, version 5.02.03).

This current study is a sub-analysis of data from a larger dataset that also involved participants that have chronic post-stroke hemiparesis. The larger study design involved the use of a robotic safety system to allow people, at risk for falls, to experience walking at very fast speeds without risk of harm if they were to lose their balance. The device has a fall harness applied at the waist. The KineAssist (KA) Gait and Balance Robotic SystemTM (HDT Global) was coupled to a Bertec treadmill (Bertec Corporation) to have similar conditions to the participants with chronic post-stroke hemiparesis. The pelvic harness of the KineAssist allowed 6-degrees of freedom at the pelvis and was used as a safety mechanism as the device would detect a loss of balance as a drop in the height of their pelvis, catching the individual in the harness system and simultaneously ceased from moving forward. The biomechanics of nonimpaired individuals walking in the KA has been described in detail in a previous study [21].

### Experimental protocol

After providing consent, the participant completed an over ground 10-meter walk test for their CWS. Then, participants were prepped for the kinematic collection and placed in the KA harness. Participants underwent a short familiarization process of walking in the KA coupled to the treadmill at comfortable speeds controlled by one of the investigators. We asked participants to decide when they felt comfortable walking in the KA and then concluded the acclimation process.

We then asked people to walk at least thirty strides at a range of eleven different speeds (0.35, 0.5, 0.65, 0.8, 0.95, 1.1, 1.25, 1.4, 1.55, 1.7, & 1.85 m/s). Order of presentation of speeds was randomized to reduce any order bias and to fulfill assumptions underlying tests for statistical inference. Rest breaks were provided as needed and, if not requested, were enforced every 5 trials.

### Data processing and analysis

The key dependent variables of this study were the SBS-FTA variability, SL variability, and MTC variability collected at the wide range of speeds. The dependent variables were gathered concurrently using the endpoint trajectory of the right foot’s 2^nd^ metatarsal head. Step kinematics of ‘foot on’ and ‘foot off’ were identified using Visual3D’s gait events algorithm using the 5^th^ metatarsal head and calcaneus’s trajectory to isolate individual steps for analysis, and the data were visually inspected to ensure accurate representation of gait events. SL was calculated by dividing the belt speed by the step frequency on a step-by-step basis. The MTC was calculated by locating the local minima of each step during the swing phase. The coefficient of variation and standard deviation (SD) were calculated for SL and MTC for each trial as below (Eqs 1 and 2).

$$SD\;=\;\surd (\frac{1}{n}\sum_{i\;=\;1}^{N}{(x}_{i}-\mu {)}^{2})$$ (1)

$$CV\;=\;\frac{SD}{mean}\;*\;100$$ (2)

Kinematic data were analyzed offline to identify if the participant transitioned to a jog or run (i.e. both feet off of the ground at the same time) for any of the speeds. Trials where the individual adopted a gait other than walking were identified, and removed from analysis.

### SBS variability calculation

**Fig 1:**
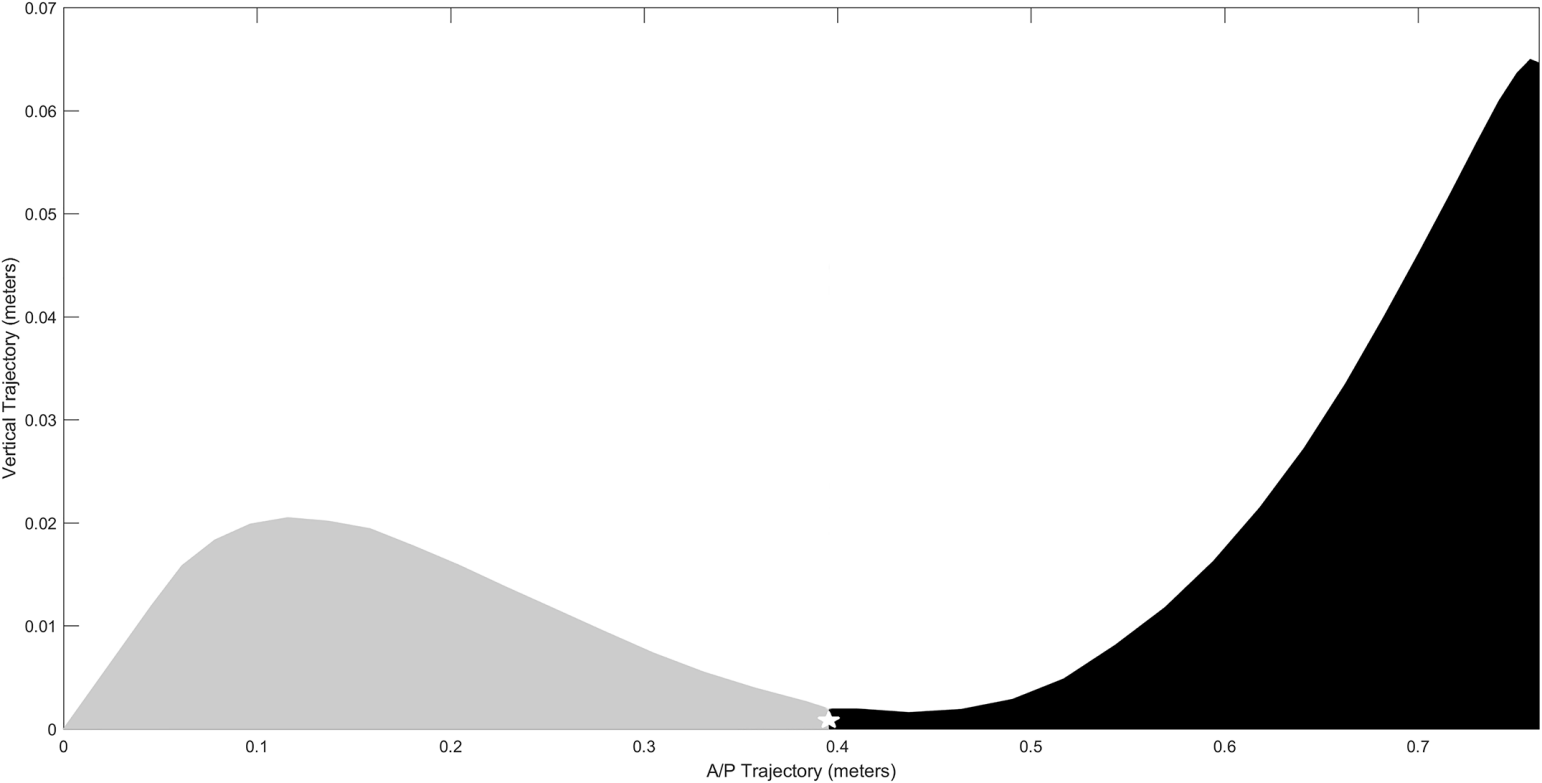
Describing creation of H_1_ and H_2_ calculation. For each step, the trajectory during the swing phase was bisected by the centroid position (white star). The area under the toe marker during swing phase from H_1_ (grey) foot off to midswing, and H_2_ (black) midswing to foot on were calculated using the “polyarea” function in Matlab. Example H_1_ SBS-FTA calculation. Step 15 area is subtracted from Step 16 area. The absolute value is the SBS-FTA difference. If Step 15 was 0.0097 m^2^ and Step 16 was 0.0101 m^2^, the SBS-FTA difference would be 0.0004 m^2^.

H_1_ and H_2_ SBS-FTA variability was calculated by bisecting the swing phase with the weighted centroid position. The weighted centroid position for each step was calculated to divide the anterior-posterior trajectory at mid-swing. The weighted centroid position was calculated by (1) locating data points from foot off to foot on, (2) the median A/P coordinate was identified and used to create the centroid position (Fig 1).

We created polygons of each of the halves of swing phase using these gait events as defining starting and ending points in the trajectory. Due to the primary interest of the combined step height and length variability during the swing phase, the areas under the footpath trajectory curve of the first half of swing (H_1_) and the second half of swing (H_2_) were calculated. They were calculated for each half using Matlab (Mathworks, R2016a) code for polyarea that returns the area of the polygon specified by the vertices of each data point in the fore-aft and vertical direction as seen in below (Eq 3).

$$\;{Matlab}^{\prime }s\;polyarea\;=\;[(x1+x2)(y1-y2)+(x2+x3)(y2-y3)+\ldots \;+(xn+x1)(yn-y1)]/2$$ (3)

We calculated the SBS-FTA changes of area for H_1_ and H_2_ by finding the absolute difference of the previous step from the subsequent step of each trial and averaging the differences of all steps (Eq 4).

SBS-FTA=1nn∑(|HHii+1-HHii|)NNii=1 (4)

### Statistical analysis

Cadence and step length data were plotted against speed to determine the extent to which participants increased speed by linearly increasing step length and/or cadence and to ensure the data looked normal. Mean descriptive statistics for all measures at all speeds were calculated. We conducted simple linear and quadratic function regressions for each dependent variable (SBS-FTA variability of the first half of swing (H_1_) and 2) the second half of swing (H_2_), 3) SD of SL and 4) MTC, and 5) CV of SL and 6) MTC variability for each participant) across speed as the independent variable. The best-fit curve was determined by first fitting a linear model and then a quadratic model. If the quadratic model added a significant increase in R^2^ (significant F change between models), compared to the lower order model, then we chose the higher order relationship. We then compared H_1_ and H_2_ SBS-FTA, MTC, and SL changes on a step-by-step basis using paired t-tests to compare the slowest speed tested to the fastest speed (without a flight phase tested) to determine which measures had the greatest magnitude of change from a slow speed to a fast speed.

Due to the observation that data were not necessarily normally distributed, we conducted Wilcoxon Signed Ranks Tests to compare the magnitude of variability of each measure captured at the average CWS of 1.4m/s [22]. The magnitude of variability is defined here as the CV for SL, MTC, H_T_ area, H_1_ area, and H_2_ area. CV for each measure was calculated by dividing each variable’s standard deviation by the mean over the course of the trial, and multiplying the value by 100. This additionally allowed us to normalize the variability to a mean value so the units were the same.

We conducted Kendall Tau-b correlation analyses on total SBS-FTA variability (H_T_: combined H_1_ and H_2_ SBS), H_1_ SBS-FTA variability, H_2_ SBS-FTA variability, step length variability, and minimum toe clearance variability across the range of speeds to establish to what extent the variables were associated with each other. We used an alpha level of .05 for all statistical tests. Corrections were used for multiple comparisons where applicable. All statistical analyses were performed using SPSS v.24.

## Results

We characterized the way that each participant increased speed on the treadmill by calculating step length and cadence. Every participant exhibited strong positive linear relationships between step length and speed (R^2^ = 0.95) and cadence and speed (R^2^ = 0.95).

We calculated standard descriptive statistics of the dependent variables across speed (Table 1). There was one individual who had multiple flight phases at 1.55+ m/s and 8 other individuals who had multiple flight phases at 1.85 m/s.

**Table 1:** Descriptive statistics of dependent variables mean(SE) across range of speeds.

N	Speed (m/s)	H_1_ SBS-FTA (10^-3^m^2^)	H_2_ SBS-FTA (10^-3^m^2^)	Mean SL (10^-1^m)	Mean MTC (10^-2^m)	SD SL (10^-2^m)	SD MTC (10^-3^m)	CV SL	CV MTC
15	0.35	0.98(0.13)	1.11(0.15)	2.94(0.13)	7.47(0.20)	2.07(0.26)	3.38(0.31)	6.92(0.76)	4.20 (0.32)
15	0.5	1.06(0.11)	1.12(0.13)	3.59(0.15)	7.59(0.23)	1.78(0.21)	3.41(0.45)	4.91(0.52)	4.65(0.53)
15	0.65	1.09(0.10)	1.22(0.12)	4.25(0.15)	7.54 (0.21)	1.59 (0.18)	3.14(0.38)	3.66(0.34)	4.33(0.56)
15	0.8	1.08(0.08)	1.33(0.10)	4.90(0.15)	7.48(0.20)	1.62(0.18)	2.85(0.23)	3.28(0.31)	3.89 (0.30)
15	0.95	1.11(0.08)	1.39(0.08)	5.30(0.13)	7.48(0.18)	1.35(1.34)	3.14(0.22)	2.54(0.25)	4.19(0.28)
15	1.1	1.42(0.12)	1.56(0.13)	5.83(0.14)	7.52(0.17)	1.65(0.18)	3.12(0.29)	2.80 (0.26)	4.34(0.39)
15	1.25	1.40(0.17)	1.81(0.16)	6.22(0.16)	7.58(0.22)	1.35(0.07)	2.97(0.21)	2.18(0.12)	4.01(0.26)
15	1.4	1.73(0.24)	2.02(0.28)	6.66(0.16)	7.60(0.20)	1.58 (0.12)	4.52(1.13)	2.38(0.16)	5.85 (0.12)
14	1.55	1.80(0.13)	1.99(0.17)	6.86(0.21)	7.66(0.14)	1.70(0.12)	3.48(0.34)	2.52(0.23)	4.52(0.42)
14	1.7	1.72(0.14)	2.00(0.21)	7.14(0.25)	7.67(0.16)	2.70(0.71)	3.57(0.23)	3.97(1.11)	4.67(0.30)
6	1.85	2.48(0.44)	2.33(0.32)	7.67(0.20)	8.28(0.40)	2.00(0.25)	4.94(0.97)	2.62(0.33)	6.65(0.15)

### Step kinematic variability measures: Model fits

We found that H_1_ and H_2_ SBS-FTA, were best fit by a positive linear relationship and were not significantly different to the quadratic model (H_1_ sig. F change = 0.2; H_2_ sig. F change = 0.26), CV SL had a negative linear fit, however, the quadratic model was a significantly better fit (CV SL sig. F change <0.01). SD SL did not have a significant fit for linear nor quadratic models. MTC increased monotonically but did not have a significant fit for any variability measure (Table 2).

**Table 2:** Model fits for variability measures.

Variable	H_1_ SBS-FTA	H_2_ SBS-FTA	SD SL	CV SL	SD MTC	CV MTC
Linear R^2^	0.66*	0.65*	0.11	0.51	0.25	0.27
Quadratic R^2^	0.73	0.74	0.49	0.68*	0.42	0.30

*Best model fit; No model fit for SD SL, SD MTC, and CV MTC

### Step-by-step magnitude

When reviewing SBS-FTA, we observed that there was more variability in the area of the footpath than with the single point SL or MTC measures. Our initial thought was that H_1_ and H_2_ SBS trajectory area variability would potentially be a superior measure of sensitivity to trajectory variability at faster speeds when compared to SL and MTC since it quantifies the changing area under the toe marker during the swing phase. Table 3 shows that H_1_ and H_2_ SBS-FTA had greater magnitude of change when comparing the slowest speed tested to the fastest speed tested on a step-by-step basis. In contrast MTC and SL step-by-step change using the slowest speed tested and the fastest speed tested was relatively low indicating that it was less sensitive to capturing speed-dependent changes.

**Table 3:** Change in MTC, SL, H_1_ and H_2_ SBS-FTA at slowest and fastest speed tested on step-by-step basis.

	Mean (x10^-4^)	Std. Deviation (x10^-4^)	Std. Error Mean (x10^-4^)	t	df	Sig.
Slow MTC vs Fast MTC	0.13	1.61	0.42	0.31	14	0.76
Slow SL vs Fast SL	1.12	7.56	1.95	0.57	14	0.58
Slow H_1_ vs Fast H_1_	-0.76	0.28	0.07	-10.39	14	0.000
Slow H_2_ vs Fast H_2_	-0.90	0.59	0.15	-5.86	14	0.000

### Step kinematic measures: Relative variability magnitude using coefficient of variation (CV)

To determine which variability measures captured the greatest amount of variability at a nominal speed (i.e. 1.4 m/s), we expressed relative variability magnitude for SL, MTC, H_1_, H_2_, and H_T_ (combined H_1_ and H_2_ area) measures using CV. H_T_, H_1_ and H_2_ had more variability relative to its mean compared to SL & MTC (Table 4). H_1_ and H_2_ had more variability compared to the H_T_ measure.

**Table 4:** Wilcoxon Signed Ranks Test: CV at 1.4 m/s for SL, MTC, H_1_, H_2_, & H_T_.

	MTC—SL	H_T_—SL	H_1_—SL	H_2_—SL	H_T_−MTC	H_1_—MTC	H_2_—MTC	H_1_—H_T_	H_2_—H_T_	H_2_—H_1_
Z	-3.41^a^	-3.41^a^	-3.41^a^	-3.41^a^	-3.41^a^	-3.41^a^	-3.41^a^	-2.04^b^	-3.41^a^	-3.12^a^
Asymp. Sig. (2-tailed)	*<0.001	*<0.001	*<0.001	*<0.001	*<0.001	*<0.001	*<0.001	0.04	*<0.001	*<0.01

^a.^ Based on negative ranks

^b.^ Based on positive ranks

*Significant after correction at p< 0.0125

**Fig 2:**
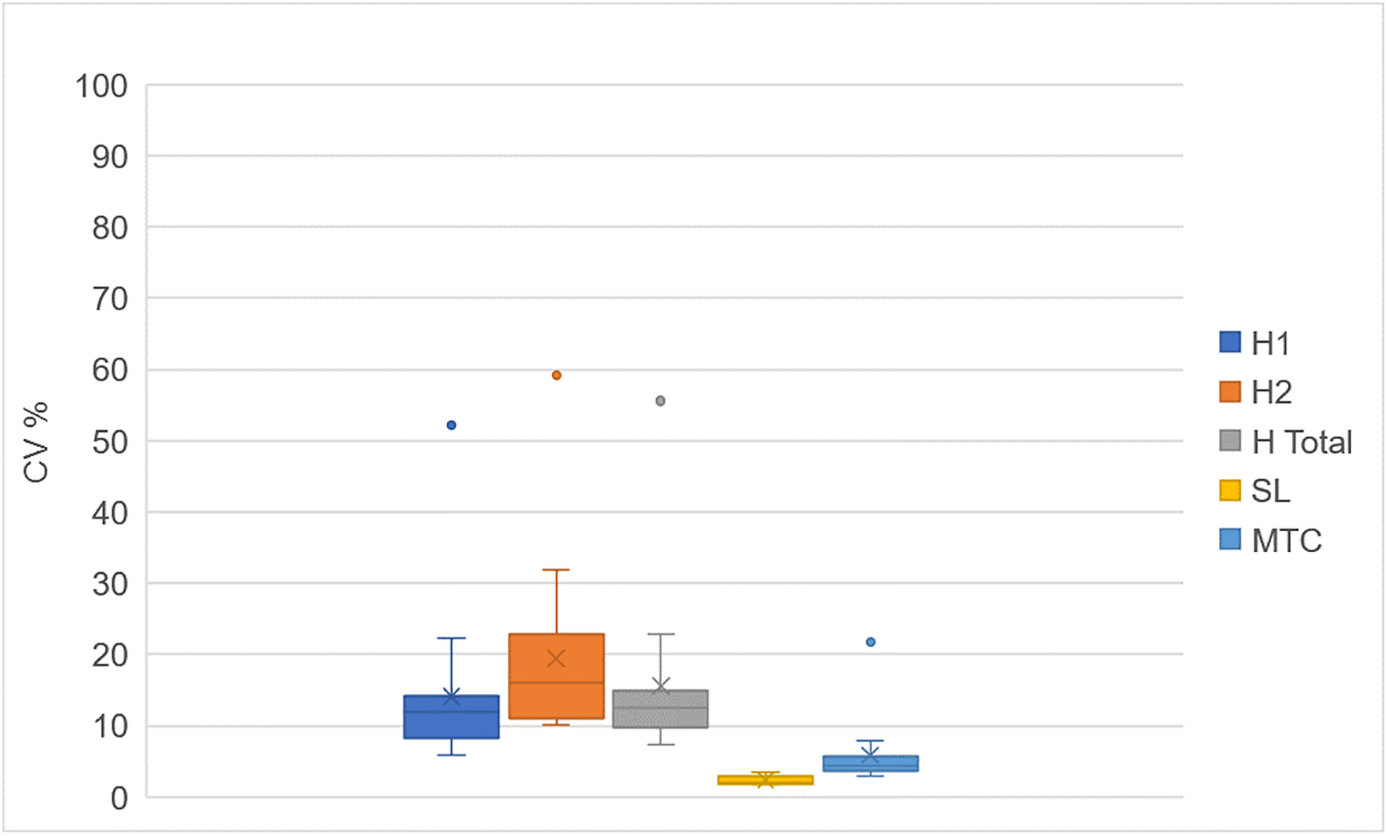
Greater CV for H1, H2 and HT for all participants compared to SL and MTC at 1.4 m/s.

At 1.4 m/sec walking speed, median foot trajectory area measures were up to six-times more variable, as H_1_ area magnitude was 11.9% (IQR = 8.34–14.1%), H_2_ area magnitude was 15.9% (IQR = 11.0–22.8%), H_T_ area magnitude was 12.4% (IQR = 9.8–15.0%), while SL magnitude was 2.0% (IQR = 1.9–2.9%), and MTC magnitude was 4.4% (IQR = 3.1–5.8%) (Fig 2).

### Step kinematic variability measures: Correlation between variability measures

To determine if the SBS-FTA variability measures co-varied with other variability measures, we conducted correlation analyses for each dependent variability measure against each other. The closer the r_τ_ was to 1, the stronger the correlation [23, 24]. SBS-FTA H_1_ and H_2_ were strongly and positively correlated with each other (r_τ_ >0.6), and weakly (r_τ_ <0.4) and positively correlated with SD of SL and MTC, and CV of MTC (Table 5).

**Table 5:** Kendall Tau-b correlation analyses between variability measures.

	SBS-FTA H_1_	SBS-FTA H_2_	SD SL	CV SL	SD MTC	CV MTC
SBS-FTA H_1_	X	X	X	X	X	X
SBS-FTA H_2_	0.70**	X	X	X	X	X
SD SL	0.36**	0.31**	X	X	X	X
CV SL	0.022	-0.02	0.57**	X	X	X
SD MTC	0.36**	0.36**	0.32**	0.18**	X	X
CV MTC	0.37**	0.36**	0.35**	0.21**	0.82**	X

** r_τ_ Correlation was significant at the 0.01 level (2-tailed).

## Discussion

The primary aim of this study was to compare a set of SBS-FTA variability measures to more commonly-used step kinematic variability measures of SL and MTC across a wide range of speeds. We hypothesized that the SBS-FTA variability would demonstrate increased variability with speed. Second, we hypothesized that SBS-FTA had a stronger curvilinear fit compared with the CV and SD of SL and MTC. Third, we hypothesized SBS-FTA would be more responsive to change in the foot trajectory at a given speed compared to SL and MTC. Fourth, SBS-FTA variability would not strongly co-vary with SL and MTC variability measures since it represents a different construct related to foot trajectory area variability. All hypotheses were supported by our results. From this we pose the following questions: 1) What does it mean that SBS-FTA increases with speed and why is it so responsive to speed? 2) Why does FTA capture the relationship with speed stronger than MTC and SL? 3) What are the implications that SBS-FTA do not strongly co-vary with SL and MTC if SL and MTC are points in the trajectory?

### Responsiveness of variability measurements with respect to speed

SL variability values observed with this experiment were like those reported in the literature. For SL SD, Sekiya, Nagasaki [3] demonstrated for speeds less or greater than comfortable walking speed (CWS), individuals increased SD SL while CV of SL [5, 6] had a small decline from 80% CWS compared to fast walking speeds.

MTC variability values, observed with this experiment, were also similar to those reported in the literature. For MTC SD does not significantly change with increasing speed according to the model, but the mean increases monotonically with speed [8, 25]. Similar to trends reported by Miller, Feiveson [8] and Ivanenko, Grasso [9], the CV values observed with this experiment followed a non-significant small U-shaped quadratic function with a minima near the average CWS for individuals. Therefore, our results appear to be a valid representations of SL and MTC variability under different speed conditions.

### H_1_ and H_2_ measures

What does it mean that SBS-FTA increases linearly with speed and why is it so responsive to speed? Increasing variability with speed could be viewed as walking performance becoming unstable or needing to transition to a new gait state [26, 27]. Conversely, others have suggested that more variability indicates that the nervous system is better able to adapt to new environmental conditions with a wider array of responses [27, 28]. SBS-FTA variability increasing with speed could be indicative of increasing instability or better adaptation. In a non-impaired population, it is more likely adaptations. SBS-FTA may have a stronger positive curvilinear fit compared to variability measurements of SL and MTC due to the multiple paths that the foot may take during the swing phase to reach the same foot fall endpoint. It is a measure that is more sensitive to changes in the footpath since it quantifies the changing area under the path under the toe marker during the swing phase. For H_1_, foot off to midswing, the initiation of swing is important since the resulting swing trajectory is thought to be directed by initial momentum of the swing [28]. Initiation of swing involves critical sensory cuing from hip flexor stretch receptors and limb unloading [29,30]. For H_2_, midswing to foot on, the foot starts to travel with maximum horizontal velocity [31] while moving anterior to the individual’s center of mass in preparation for initial contact. A new base of support for the subsequent step is established and the dynamic stability with respect to the position and velocity of the center of mass is reestablished [32, 33]. In the case of the non-impaired nervous system, could it be possible that the nervous system doesn’t care about the path that the foot takes so long as the foot lands at the destination?

### Relative variability magnitude measure

Why does SBS-FTA capture more variability relative to its mean compared to SL & MTC at a given speed? SBS-FTA captures the changing area under the toe marker, while SL and MTC are singular spatially tied data points that act independent of each other. SBS-FTA has more possible changes in area solely based on the number of trajectories that the foot can travel through on a step-by-step basis allowing us to observe more variability relative to a mean value. This may be indicative of observing more variability in the foot trajectory that the other measures may not capture.

### Degree of relationship between variability estimators: Correlation

Is SBS-FTA a different construct? We hypothesized that SBS variability would not strongly co-vary with SL and MTC variability measures. We observed that SBS H_1_ and H_2_ only weakly positively correlated with SD of SL and MTC and with CV of MTC indicating that the H variables appear to capture a unique construct of foot trajectory area variability. The combination of the measures not strongly covarying with H_1_ and H_2_, and H_1_ and H_2_ SBS variability increasing with speed, indicates that the numerous trajectories possible are not completely dependent on MTC nor SL.

### When is each measure useful for representing kinematic variability

Is there a case where one of the variability measures would be better to use than the others? H_1_ and H_2_ SBS-FTA variability may be a better measure to use when examining changes in variability at faster speeds when compared to SL and MTC variability. MTC and SL provide information about points along that trajectory, but they do not provide information on the behavior of the continuous trajectory of the foot on a step-by-step basis. SBS-FTA may be a tool to acquire more sensitive information about endpoint control of the foot. Additionally, while the MTC and step length variability may be appropriate for non-impaired individuals, for individuals poststroke MTC is often not systematically identifiable and the different areas that the trajectory may generate for individuals poststroke are not accounted for [34, 35]. Though, the SBS trajectory area variability measure is more sensitive to foot trajectory variability, it is slightly more complicated to calculate, and SL works well to measure the variability for non-impaired individuals.

### Limitations

There are a few study limitations that should be noted. The H_1_ and H_2_ SBS foot trajectory area variability measure we used is a relatively new measure, which captured the change in area under the trajectory curve of the toe marker during the swing phase. We developed this measure in order to combine the variability of foot trajectory in both the vertical and fore-aft directions. Breaking the trajectory into halves allowed us to observe the combined fore-aft and vertical trajectory during the initial and terminal swing phases. Additionally, the data that was used for this analysis was only in the sagittal plane and did not account for medial-lateral movement. Due to the primary interest of comparing H_1_ and H_2_ SBS foot trajectory area variability to SL and MTC, we decided that it was more appropriate to utilize only sagittal plane data.

## Conclusions

SBS-FTA variability was sensitive to change with faster speeds, captured a relationship that the majority of the other measures did not demonstrate, and did not co-vary strongly with other measures that are also components of the foot trajectory. SBS-FTA foot trajectory area variability was sensitive to change with faster speeds, captured a relationship that the majority of the other measures did not demonstrate, and did not co-vary strongly with other measures that are also components of the foot trajectory. Future studies may be performed to determine if these measures may be useful in identifying people at risk for loss of control at faster speeds (i.e. older individuals at high fall risk and people with neurologically impaired gait, such as poststroke and Parkinson’s disease).

## Abbreviations

CV: coefficient of variation
CWS: comfortable walking speed
H1: foot off to midswing
H2: midswing to initial foot contact
IQR: interquartile range
KA: KineAssist Gait and Balance Robotic System
MTC: minimum toe clearance
SBS-FTA: step-by-step foot trajectory area
SD: standard deviation
SL: step length
UAB: University of Alabama at Birmingham
